# Kailo: a systemic approach to addressing the social determinants of young people’s mental health and wellbeing at the local level

**DOI:** 10.12688/wellcomeopenres.20095.1

**Published:** 2023-11-13

**Authors:** Tim Hobbs, Ediane Santana De Lima, Dickon Bevington, Cristina Preece, Kate Allen, Pia Barna, Vashti Berry, Thomas Booker, Karuna Davies, George Davis, Jessica Deighton, Leanne Freeman, Peter Fuggle, Ellen Goddard, Tamsin Greene Barker, Julie Harris, Amy Heather, Mary-France Jardiel, Krishna Joshi, Megan Keenan, Laura Kennedy, Tamanna Malhotra, Anna March, Steve Pilling, Martin Pitt, Katie Potter, Nirandeep Rehill, Jenny Shand, Rachel Surtees, Peter Fonagy

**Affiliations:** 1 Dartington Service Design Lab, Buckfastleigh, England, TQ11 0EE, UK; 2 Anna Freud Centre, London, England, N1 9JH, UK; 3 University of Exeter, Exeter, England, EX4 4PY, UK; 4 UCLPartners, London, W1T 7HA, UK; 5 Research Department of Clinical, Educational and Health Psychology, University College London, London, England, WC1E 6BT, UK

**Keywords:** Mental Health, Wellbeing, Social Determinants, Stakeholder Involvement, Co-design

## Abstract

The mental health and wellbeing of children and young people is deteriorating. It is increasingly recognised that mental health is a systemic issue, with a wide range of contributing and interacting factors. However, the vast majority of attention and resources are focused on the identification and treatment of mental health disorders, with relatively scant attention on the social determinants of mental health and wellbeing and investment in preventative approaches. Furthermore, there is little attention on how the social determinants manifest or may be influenced at the local level, impeding the design of contextually nuanced preventative approaches. This paper describes a major research and design initiative called Kailo that aims to support the design and implementation of local and contextually nuanced preventative strategies to improve children's and young people’s mental health and wellbeing. The Kailo Framework involves structured engagement with a wide range of local partners and stakeholders - including young people, community partners, practitioners and local system leaders - to better understand local systemic influences and support programmes of youth-centred and evidence-informed co-design, prototyping and testing. It is hypothesised that integrating different sources of knowledge, experience, insight and evidence will result in better embedded, more sustainable and more impactful strategies that address the social determinants of young people’s mental health and wellbeing at the local level.

## Background

### Need and inequalities

In general, the mental health of children and young people is deteriorating: the prevalence of many mental health disorders is on the rise; wellbeing is decreasing; and inequalities in mental health are widening for some groups ( Castelpietra *et al*., 2022; Newlove-Delgado *et al*., 2022).

The picture is, of course, more nuanced than this. There are some areas of progress, such as a modest reduction in youth suicide and substance misuse rates, and the introduction of waiting time standards for accessing first episode psychosis and eating disorder services for young people ( NHS England, 2021; NHS England, NICE and NCCMH, 2016; Office for National Statistics, 2022).

Yet generally speaking, the mental health and wellbeing of young people is deteriorating and the impact of this on life-course trajectories and for society remains a cause of significant concern to practitioners and policy-makers; with some describing it as being ‘in crisis’ ( Gunnell *et al.*, 2018).

### Treatment and prevention

Over the last two decades, there has been a substantial investment in mental health services, treatment responses, and research ( Cohen, 2017). This has been, in part, driven by rapid and productive advances in the life sciences which have helped inform approaches to early identification, design, and implementation of targeted and universal interventions ( HM Government, 2021).

However, much of this investment is heavily skewed towards individuals, treatment responses, narrowly defined health outcomes, and mono-causal assumptions ( Knapp & Wong, 2020). While significant positive advancements have been made in the treatment of mental health difficulties, current service provision for young people is almost universally described as overwhelmed, inadequately funded, and lacking capacity to meet rising demand ( Lennon, 2021).

If advancement and investment in the treatment of mental health difficulties are judged to fall short, then advancements and investments in the prevention of poor mental health may be deemed wholly inadequate.

The sheer scale of need and the treatment gap ( Kohn *et al*., 2004) means that, arguably, attempts to develop and deliver many specialised treatments require an extensive and narrow funnelling of finite resources to remedial responses (at the national and local level). This, in the language of systemic archetypes, may be considered a short-term ‘fix that fails’ ( Hulme *et al*., 2022; Wolstenholme, 2003): whilst necessary, treatment only responds to surface-level manifestations of need without addressing the underlying systemic and structural drivers that perpetuate the issues. This, in turn, may further drain the finite pool of resources away from health promotive and preventive efforts, further compounding the need. Specialised treatments that rely on specialised treaters (numbers of whom cannot easily be scaled-up, especially commensurate to the extent of the existing treatment gap) paradoxically risks compounding inequality of access to help, which is in and of itself accepted as a key social determinant of mental health in a population ( Compton & Shim, 2015).

So, whilst a continued and increasing investment is required in relation to the treatment of mental health disorders, this must also be accompanied by significant investment and redoubling of efforts to design, test and deliver at scale effective prevention and population-level mental health promotion approaches ( Mc-Daid & Park, 2022; Muñoz *et al.*, 1996).

### The social determinants of young people’s mental health: a systemic issue

Concordant with calls for an increased emphasis on prevention, there has been growing attention to the social determinants of population health, including mental health. It is now widely acknowledged that a range of demographic, neighbourhood, social, cultural, economic, and environmental influences interact to affect young people’s mental health (and exert influence upon the access to, and efficacy and impact of, services and systems of support ( Compton & Shim, 2015; Lund *et al*., 2018). These various social determinants of mental health reciprocally drive, and are driven by, social inequities, poverty, and deeply entrenched systemic discriminations ( Alegría *et al*., 2018).

As such, mental health may be considered a ‘wicked problem’ ( Hannigan & Coffey, 2011) with multiple interacting synergies: it is no more attributable to a single causal agent (the rapid expansion of access to social media, for instance) than it is to, say, an inflationary redrawing of diagnostic boundaries that pathologises ordinary human distress ( Lee, 2014) or the lowering of culturally-sanctioned thresholds for help-seeking (with the moral opprobrium that may accompany such observations ( Thomas *et al*., 2018)).

Given the multitude of interacting influences, we argue that young people’s mental health and wellbeing must therefore be considered a ‘systems issue’ ( Cohen, 2017; Fried & Robinaugh, 2020; Hodges *et al.*, 2012; Meadows, 2008). This perspective considers mental health and wellbeing as a dynamic state that varies over time and is influenced by the interactions of these wider social determinants.

It follows that efforts to improve young people’s mental health require a nuanced understanding of local influences, and a multi-pronged approach to addressing locally relevant, high-impact leverage points ( Betancourt *et al*., 2011; Groark *et al.*, 2011; Salam *et al*., 2022; Ungar & Theron, 2020).

### Varying manifestations at the local level

In wider fields of public health, systemic intervention efforts tend to focus on macro-system policy levers such as poverty, economic inequality, employment, housing, and transport ( Marmot, 2020). There is significant potential for impact operating at this level, although sustained policy change is challenging and highly politicised.

We argue that as well as considering the macro-influences, it is also important to take a more nuanced local perspective, exploring how the social determinants of mental health are manifest at the micro/local level. The ways in which the social determinants influence young people’s mental health will vary depending on local context, individual circumstance, and their local interactions ( Alegría *et al*., 2018). To take an over-simplified example: in an inner-city urban environment, poverty may contribute to overcrowded housing, in turn, driving young people into potentially unsafe neighbourhood environments, whereas in a rural context similar levels of poverty may manifest as limited access to transport, isolation and reduced opportunities. These different risks or contexts may, in different ways, lead to the same outcome, e.g., poorer mental health (i.e., the concept of equifinality ( Cicchetti & Rogosch, 1996; Fried & Robinaugh, 2020)).

Understanding and designing preventative responses in a contextually nuanced way is critical if we are to meaningfully affect underlying dynamics over time. As such, we argue that as well as considering the macro-systemic influences it is also important that we take a more nuanced local perspective, exploring how the social determinants of mental health are varyingly manifest at the micro/local level, and from this local understanding, design and implement contextually relevant preventative responses.

### Existing frameworks for understanding local needs and guiding prevention efforts

There are a wide range of different approaches by which local leaders and community partnerships seek to understand local needs and context and, in turn, design and implement strategies, policies and practices to improve population mental health and wellbeing. Local needs and context may be understood, for example, via community-led and participatory action research ( Burgess *et al*., 2022), quantitative needs assessments or school / community-based epidemiological surveys ( Connors *et al*., 2015; Hughes *et al.*, 2022), local stakeholder and asset mapping ( Duncan *et al*., 2021; Public Health England, 2018) and the mapping of local system dynamics ( Noubani *et al*., 2020; Stansfield *et al*., 2021). Local action or intervention may result from local co-design efforts ( O’Brien *et al.*, 2021; Tindall *et al*., 2021), social action and community organising ( Bolton *et al*., 2016), through to strategic commissioning of new or existing practice, or evidence-based prevention or early intervention programmes ( Boaz *et al*., 2019).

Over the last two decades, a number of structured ‘strategic prevention frameworks’ or ‘operating systems’ have been designed, tested and implemented ( National Research Council (US) and Institute of Medicine (US) Committee on the Prevention of Mental Disorders and Substance Abuse Among Children, Youth, and Young Adults, 2009). These incorporate a series of structured steps, typically including: (i) identification of local prevention needs based on existing or new data; (ii) forming local partnerships and governance structures to identify priorities and build local capacity and momentum; (iii) identification and implementation of evidence-based programmes and practices; and (iv) ongoing monitoring, evaluation and learning. Examples include broad frameworks or guides (e.g., the US SAMHSA Strategic Prevention Framework, 2019) through to more structured approaches (such as Communities that Care ( Fagan *et al*., 2018), PROSPER ( Spoth *et al*., 2013) and Getting to Outcomes ( Chinman *et al*., 2008). These prevention frameworks have, in some contexts, demonstrated positive impacts on outcomes ( Brown *et al*., 2011; Crowley *et al*., 2011; Oesterle *et al*., 2018; Spoth *et al*., 2017).

Key features and strengths of these approaches include:

- Collection and synthesis of robust local data to help make the case for local action and identify priorities ( Arthur *et al*., 2002; Axford & Hobbs, 2010).
- Development of local partnerships, governance and system leadership arrangements to guide decision-making ( OECD, 2019).
- Drawing upon repositories of evidence-based programmes (EBP) or practices that have been demonstrated through rigorous experimental evaluation to improve outcomes ( Burkhardt *et al*., 2015; Catalano *et al*., 2012).

However, we argue there are some important limitations or inhibitors to impact at scale for such prevention frameworks, particularly when considering the systemic nature of the social determinants of young people’s mental health and wellbeing. The following critiques do not amount to a rejection of the approach, but rather point to ways they may be further optimised:

- Local epidemiological data and profiles of risk and protective factors may be valuable in identifying specific areas or need or strength, but alone they can obscure the systemic influences, dynamics and inter-dependencies of specific local influences ( Patel & Goodman, 2007).
- Local partnership and governance arrangements - whether situated within local government, health systems or local community forums - tend to concentrate decision-making within existing and dominant power structures (and not often with young people and/or lesser-heard or marginalised voices within communities) ( Anderson-Carpenter *et al*., 2017; Chilenski *et al*., 2023; Fagan *et al*., 2019).
- A reliance on existing evidence-based programmes (EBP) may: (i) be undermined by the increasingly recognised challenge of replicating the impact of EBPs in new contexts ( Shidhaye, 2015); (ii) reduce availability of provision options, based on limited EBP provider availability in the local area ( Harvey & Gumport, 2015); (iii) miss opportunities to build local ownership, alongside disenfranchising or critically undermining relationships and trust with local providers of similar, albeit not so strongly (formally) evidenced practice ( Mullen & Streiner, 2004); (iv) stifle local innovation ( Dryden-Palmer *et al.*, 2020); and (v) not adequately reflect the nuance of local needs or context ( Baumann, 2010).

As such, we hypothesise that the impact and uptake of prevention frameworks may be further enhanced if they are better able to: (a) move beyond narrow conceptualisations of risk and protection and also consider and address the systemic nature and social determinants of young people’s mental health; (b) elevate and integrate youth and community voices when setting local priorities; and (c) more effectively balance evidence-informed practice with local innovation and co-design. It is in response to these gaps and opportunities that we have designed and plan to implement and test ‘Kailo’ ^1^: a new systemic prevention framework to address the social determinants of young people’s mental health at the local level. 

## Aims and objectives

Our long-term vision is to demonstrably improve, at the local level, youth mental health and wellbeing outcomes via the design and implementation of preventative approaches that address contextually relevant social determinants of health.

Our objectives are:

1) To create a prevention framework (Kailo) that:
  a) Helps local public system and community partnerships better understand how the social determinants of young people’s mental health and wellbeing manifest at the local level;
  b) Elevates youth and community voice in determining priorities for change;
  c) Highlights inequalities in experiences and outcomes as a focal point for change;
  d) Brings young people, community partners and professionals together in co-designing systemic and evidence-informed strategies to address these social determinants, inequalities and improve young people’s mental health and wellbeing; and
  e) Integrates these priorities and designs into local strategic planning and commissioning to enable sustained change.
  2) To implement this framework in two distinct geographical contexts, and through practice-based learning and developmental evaluation seeking to explore what works, for whom, under what circumstances, and how ( Wong *et al*., 2016);
  3) Incorporate learning into a refined, replicable and locally owned framework that is adopted in new contexts and evaluated for impact on population-level mental health and wellbeing outcomes.

These objectives are underpinned by the following research questions:

- RQ1: How does Kailo function as an initiative? Why and for whom?
- RQ2: How is Kailo received in a local context and what conditions are necessary for place-based systems change to be achieved through Kailo?
- RQ3. What is the impact of Kailo, in relation to the alignment and coordination of local resources and systems of support (and how does this vary by context)?
- RQ4. What is the impact of Kailo in relation to young people’s mental health and wellbeing outcomes and associated inequalities (for whom, and how does this vary by context)?
- RQ5. What is required in order to effectively scale the Kailo framework?

### Kailo Framework

Kailo is a prevention framework designed to help local community and public system partnerships elevate the voice of young people in designing systemic, evidence-informed strategies and interventions that systemically address the social determinants of young people’s mental health and wellbeing in the local context.

Kailo is a framework that operates across three main phases:

1. **Early Discovery:** including building strong and trusted local partnerships, understanding what matters locally, and community forming around shared priorities.
2. **Deeper Discovery and Co-Design**: A structured method of youth-centred co-design that takes a systemic, equitable and evidence-informed approach.
3. **Prototyping, Implementation and Testing**: A process of embedding designs into local infrastructures and iteratively testing and refining them.

Within each phase is a series of tools and structured research and design activities (see Table 1). These include system mapping methods, co-design, data (through existing administrative and new local epidemiological data) and different forms of evidence (practice- and lived experience evidence alongside rapid reviews of existing research and robust evaluations).

**Table 1.** Describes the different stages of the Kailo programme, their aims, activities, intended outcomes and indicators of success.

Phase	Aim	Inputs and Prerequisites	Activities	Intended Outcomes	Key Indicators of Success
**EARLY DISCOVERY**	**Build strong and trusted relationships with local partners.**	Openness of local partners to embrace a public health approach to mental health promotion. Resources to enable equitable engagements of young people and community partners.	***Ecosystem and Power Mapping:*** *(a) Snowballing key stakeholders and influencers ( Leventon *et al.*, 2016); (b) identifying lesser-heard or marginalised voices ( Pratt, 2019); (c) compiling local strategies and initiatives.* ***Relationship Building:*** *(a) mutual value activities; (b) mutual value agreements for working together ( Rycroft-Malone *et al.*, 2015)*.	Creation of trust and conditions for local ownership	Number and diversity of local community partners involved in the Early Discovery workshops and events.
	**Understand what matters, locally.**	Capacity for Kailo team and/or partners to undertake and synthesise discovery research. Data sharing agreements.	***Social Determinants Lens:*** *(a) Evidence summaries; (b) Systems map of the social determinants of young people’s mental health (causal loop diagrams ( Sharma *et al.*, 2021)*. ***Local Data:*** *(a) Synthesis of existing data on mental health and local influences; (b) New epidemiological school-based data via #BeeWell Survey ( Black *et al.*, 2023)*. ***Local Insight Generation:*** *(a) qualitative engagements about ‘what matters locally’, from young people, community representatives and local system practitioners and leaders ( Schiavo, 2021)*. ***Sense-making:*** *(a) thematic analysis and clustering ( Braun *et al.*, 2019); (b) identification of ‘opportunity areas’ translated into ‘how might we’ questions*.	Shared understanding of local social determinants of young people’s mental health and wellbeing.	Number of engagements involving local partners and young people. Diversity of local community partners and young people involved. Opportunity areas surfaced are related to social determinants identified in wider literature and contextually relevant. Opportunity areas surfaced represent what is needed and wanted locally.
	**Forming communities around youth- and community- centred priorities**	Building upon prior foundational relationship building. Time and resources to facilitate and further engage local partners.	***Playbacks:*** *(a) sharing back emerging themes and learning; (b) iterative refinement and validation ( Santana de Lima *et al.*, 2023, unpublished report)*. ***Prioritising Opportunity Areas:*** *(a) focus groups; (b) voting (or Delphi or nominal group technique ( McMillan *et al.*, 2016)*. ***Community Forming around priorities:*** *(a) Engaging community partners around priorities; (b) youth peer researcher recruitment ( Spuerck *et al.*, 2023); (c) establishing a ‘larger circle’ of supporters*.	Communities formed around shared priorities for addressing local social determinants of young people’s mental health and wellbeing.	Number of engagements involving local partners and young people. Number and diversity of local partners interested in Kailo formalised community partner roles. Prioritised opportunity areas are related to social determinants identified in wider literature and contextually relevant.
**DEEPER DISCOVERY AND CO-DESIGN**	**Co-designing systemic responses to social determinants**	Local commitment to youth-centred co-design Time and resources to engage and support young people and co-design teams.	***Co-design Team Formation:*** *(a) formation of youth and community ‘small circle’ co-design teams ( McKercher, 2020, pp.1–225); (b) mutual value agreements; (c) building trust and relationships ( Clarke *et al.*, 2021)*. ***Deeper systemic Discovery:*** *(a) refinement of Opportunity Area definition; (b) participatory group model building ( Siokou *et al.*, 2014); (c) identification of systemic intervention or leverage points ( Glenn *et al.*, 2020)*. ***Evidence Reviews:*** *(a) Production of evidence briefings; (b) rapid realist review ( Saul *et al.*, 2013); (c) Youth and Community Research into topic areas ( McCabe *et al.*, 2023)*. ***Youth-centred Co-design and Theories of Change:*** *(a) Design Thinking ideation ( Adikari *et al.*, 2016); (b) intervention design (including associated theories of change); (c) determination of necessary implementation conditions, resources and requirements*.	Locally owned, evidence-informed designs addressing the local social determinants of young people’s mental health and wellbeing.	Number of co-design sessions involving community partners and young people Diversity of actors engaged in the circles of co-design Strategies developed in co-design and prototyping sessions: Have a youth and community voice-centred approach; Address/consider contextually relevant social determinants; Are based on young people and community members views and key needs; Are focused on prevention rather than services interventions; Challenge local inequalities related to the prioritised opportunity areas; Are feasible and sustainable within the constraints of local assets and resources; and Are informed by extant evidence on what works to support young people’s mental health.
**PROTOTYPING, IMPLEMENTATION AND TESTING**	**Local system integration, prototyping and iterative refinement**	Engagement of local system leaders. Human and/or financial resources to support implementation. Research skills and capacity to support early-stage monitoring and testing.	***Playbacks to system-leaders and communities:*** *(a) Review by local partners to enhance likelihood of impact and sustainability; (b) Identification of implementation opportunities, partners and enablers*. ***‘Low fidelity’ prototyping and testing:*** *(a) prototyping via system dynamic simulation modelling ( Darabi & Hosseinichimeh, 2020); (b) small-scale implementation; (c) rapid-cycle testing ( Green *et al.*, 2021); (d) refinement of theories of change and service/practice/policy refinements required for sustained and impactful implementation*. ***‘High fidelity’ sustained implementation:*** *(a) embedding into local infrastructures; (development of data systems and monitoring, evaluation and learning frameworks*.	Interventions that are locally embedded. Improved youth mental health and wellbeing outcomes.	Strategies are implemented in the local contexts. Community partners are confident in their ability to implement local strategies. Robust evidence of intermediate and longer-term impact on young people’s mental health and wellbeing outcomes.

Implementation of the Kailo Framework and the activities described in Table 1 are underpinned by a set of guiding principles for those implementing it:

- Working collaboratively with the people and communities that will be impacted;
- Adding value and building capabilities, rather than being extractive or burdensome;
- Recognising bias and inequalities and striving to reduce them;
- Making space for reflection and learning throughout.

The integration of these principles and different sources of insight and knowledge through a systemic lens is intended to inform a contextually nuanced set of intervention points and local priorities with potential for impact. In turn, evidence-informed co-design approaches are hypothesised to result in a coordinated portfolio of high-leverage local interventions that, in turn, will lead to intermediate community-based outcomes and longer-term improvements in adolescent mental health and wellbeing (by addressing the locally relevant social determinants of health).

### Audiences and roles

**Figure 1.**
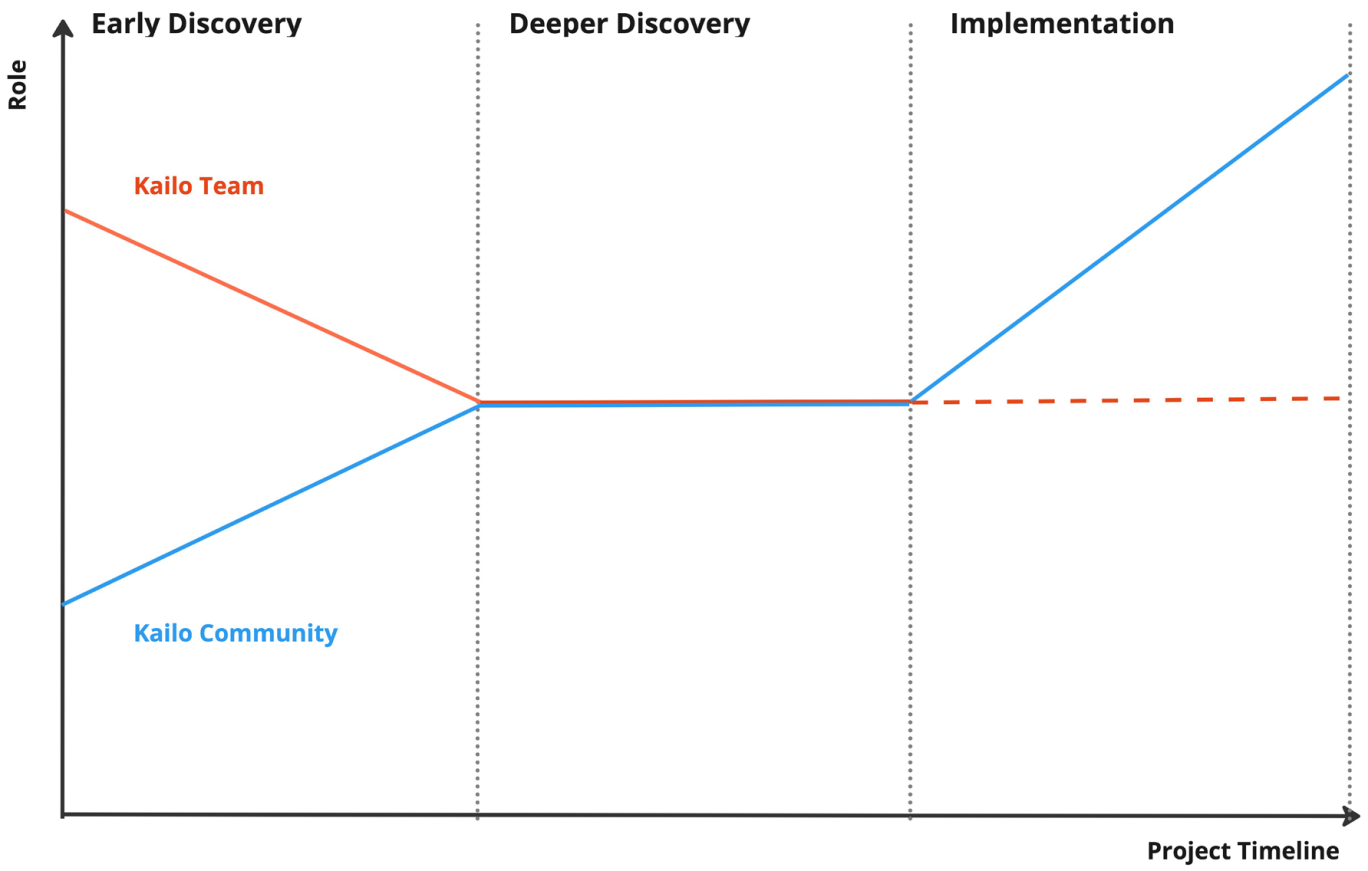
Shifting ownership of Kailo over phases. This image demonstrates how the Kailo team hopes to shift their role through the different phases of the Kailo Programme. The Kailo Community (blue), which includes local community members and young people, should become the main drivers of the Kailo programme locally, with the support of the Kailo team that initially was steering the project.

The Kailo Framework is primarily intended for use by local authority and integrated health partnerships (such as Integrated Care Partnerships in England, or Health and Social Care Partnerships in Scotland) working in partnership with local communities. The framework and phases are designed to gradually shift ownership of the work in a local area from a facilitating Kailo team to the local community partnership (as illustrated in Figure 1). This relates to one of the underpinning principles (i.e., to add value and build local capabilities).

Understanding, prioritising, co-designing and testing local responses to the social determinants of young people’s mental health is a complex task, requiring a wide range of activities - as illustrated in Table 1. Kailo is designed as a ‘modular’ approach in that different activities may be undertaken (or may have already been undertaken) in a local area in different ways by different local stakeholders or actors, to varying degrees of intensity or depth. Kailo acts as a framework or guide to prioritising, designing and testing local approaches to the social determinants of young people’s mental health and wellbeing, with an accompanying set of tools and methods which can be adopted as required.

It is our hypothesis that each element is required, and that the rigour and depth of each stage will be associated with greater buy-in and likelihood of impact, but that all stages need not necessarily be led by a central Kailo team. For example, if robust existing local data and analysis of the social determinants of young people’s mental health has already been undertaken by local partners, or local community partnerships are already well established around local priorities associated with the social determinants of young people’s mental health, then such activities or infrastructures may (and indeed should) be drawn upon, rather than replicating existing efforts.

### Kailo v1.0: Initial implementation sites

An initial version of the Kailo Framework (v1.0) is being implemented in two intentionally distinct geographical contexts Northern Devon (a rural/coastal region in the Southwest of England) and in the London Borough of Newham (a densely populated and highly diverse urban context). These two contrasting implementation contexts were identified in order to test the Kailo Framework’s ability to surface locally and contextually specific manifestations of the social determinants of young people’s mental health, and in turn inform locally nuanced and relevant policy and practice responses.

Conclusion of the ‘Early Discovery’ phase in each site has, as intended, resulted in local priorities that reflect contextually nuanced manifestations of the social determinants of young people’s mental health, whilst also surfacing and recognising some cross-cutting priorities. For example, in Northern Devon a lack of diverse opportunities for young people and a diminished sense of identity and belonging has been prioritised, whilst in Newham priorities related to community safety and discrimination have emerged. Yet, priorities around mental health-related norms and expectations emerged across both sites. This suggests promise in relation to the Kailo Framework’s ability to bring into focus locally relevant manifestations of the social determinants of young people’s mental health.

### Evaluation framework and Kailo v2.0

It is intended that insights from early implementation and the developmental evaluation of v1.0 of the Kailo Framework in the two pathfinder areas will inform a refined version of the framework (v2.0) that can be implemented in additional sites. These learnings will also inform wider replication and the subsequent contributory impact evaluation to assess how the framework contributes to improvements in adolescent mental health, changes in the wider social determinants, and local shifts in commissioning practices.

Given the complexity of the Kailo Framework, a developmental realist-informed evaluation will be conducted in the two pathfinder sites ( Kennedy *et al*., 2023, in preparation). This evaluation will move beyond the binary question of effectiveness ( Raine *et al*., 2016) and seek to explore what works, for whom, under what circumstances, and how. As such, a developmental realist-informed evaluation will be conducted ( Pawson & Tilley, 1997; Westhorp, 2014). This will investigate how and why Kailo works, for whom, and under what circumstances. This mixed-methods evaluation will engage key members of the Kailo consortium, local stakeholders, and young people who have interacted with Kailo in the pilot sites. The initial phase incorporates a rapid realist synthesis, interviews with key informants, observations, and document analyses to formulate the initial programme theory ( Jagosh, 2019; Manzano, 2016). The second phase will employ semi-structured interviews, focus group discussions, observations, and analyses of routinely collected data to test the initial programme theory ( Manzano, 2022). The final phase will employ focus group discussions to refine and consolidate the initial programme theory ( Shearn *et al.*, 2017). The developmental nature of this evaluation will facilitate sharing of feedback to improve programme implementation and support continuous learning and adaptation ( Gamble, 2008).

As the Kailo framework matures and is scaled to new sites, a summative impact evaluation will be designed and implemented, addressing research questions related to impact on sub-group and local population-level outcomes and inequalities.

### Inherent tensions, anticipated and early challenges and how Kailo is responding

In this section, we outline six key anticipated challenges, some of which are being experienced in the early stages of implementation, and how Kailo is responding.

First, there has been a legacy of national and local re-organisation and change initiatives that are not sustained. It is commonplace in local government and community partnerships for there to be history of change and reorganisation, which may not lead to tangible or observed change to community outcomes or experience ( Alderwick *et al.*, 2022). Kailo, as another initiative, risks perpetuating such change fatigue. As such, the principle of adding value is critical. Rather than acting as another initiative on top of others, Kailo is positioned in local areas as feeding into and bolstering existing initiatives and policy directives. This may include seeking to build capacity, resources and precision to hotspots of pre-existing community-based practice, social action and alliances (where sufficiently aligned), as well as integrating priorities and emerging designs into local strategies and existing governance arrangements. 

Second, early experiences of implementation of the Kailo framework suggest a strong pull from senior leaders and commissioners towards focusing on service and treatment responses - the status quo - rather than a preventative focus centred on the social determinants of mental health ( Mc-Daid & Park, 2022). This is particularly expressed from public system leaders, commissioners and practitioners, albeit much less so from young people and community partners and representatives. To mitigate against this risk, in most of our communications, articulation of aims and interactions in local areas, we consistently and routinely emphasise the intentional focus on prevention and the social determinants of young people’s mental health and wellbeing ( Faust & Menzel, 2011; World Health Organisation: Department of Mental Health and Substance Dependence, 2002). We are also at pains to communicate this is not to say that further coordination and investment in treatment services is not critical, but that this is not the role for Kailo (although insights and learning from early discovery phases can support and make the case for such investments). 

A third tension is the systemic focus of Kailo, the iterative and emergent approach to discovery and co-design ( Pailthorpe, 2017), and the evaluative frame of considering contribution in relation to context (oftentimes at odds with positivist causal assumptions and attribution) ( Nyein *et al*., 2020). These tensions are expressed less-so in local communities, but more so within the academic and research contexts (as well as within our own multi-disciplinary research consortium). This speaks to wider debates in the field about what types of evidence are valued ( Glasgow & Emmons, 2007; Rycroft-Malone *et al*., 2004).

Fourth, as introduced above, is the tension in considering what types of evidence are valued, by whom, and in what contexts ( Beames *et al*., 2021). It is not uncommon for lived/living experience, youth and community voice to be considered less rigorous, valuable or at odds with other forms of evidence, such as quantitative data or more generalised evidence ( O’Leary & Tsui, 2022). Within Kailo we are seeking to break down such false divides, through generating and surfacing different sources of insight and viewing points on specific issues in different ways, that are proportionate and appropriate to the specific questions being explored. For example, youth and community voices can explore and challenge the generalisability of existing evidence to local context, whereas existing research evidence may challenge poorly substantiated beliefs and help strengthen emerging intervention designs (based on what has been tried and tested elsewhere). It may be that different sources of insight and evidence can be aligned and reconciled, or it may transpire that they are in more fundamental opposition. Yet what Kailo seeks to advance a dialogue between multiple ‘positions’ in order to advance at least a shared understanding and respect of these different viewing points so that ‘epistemic trust’ and an openness to differing sources and forms of knowledge, insight and learning may be built ( Fricker, 2007; Schröder-Pfeifer *et al*., 2018; Tuomela, 2007 ).

Fifth, we anticipate ongoing tensions in relation to where decision-making power resides, and how such power is shared or transferred ( Joseph-Williams *et al.*, 2014). Typically, power and decision-making for setting regional and local priorities, strategies and associated resource allocation sit with senior leaders within public systems (often with wide and geographically distributed remits). This inevitably means that decision-making may not closely reflect a nuanced understanding of needs, contexts and what matters locally ( Seixas *et al*., 2021). Conversely, grassroots, youth or community-based designs may be removed or disconnected from the policy, fiscal and commissioning constraints. This speaks to the need to better connect and bridge local public system decision-making and design with the assets, insights and power that resides within local communities ( Local Government Association and NHS Clinical Commissioners, 2020). This is something we are attempting to do with Kailo, and the way in which ‘small circle’ co-design teams are nested within ‘big circles’ of community and public system leadership. Our early implementation experiences suggest how critical it is to carefully nurture and connect local relationships and build trust within and between different stakeholder groups - something echoed in wider research ( Frerichs *et al*., 2017; Metz *et al*., 2022 Vangen & Huxham, 2003; Wilkins, 2018).

Finally, as we embark on the co-design phases of the Kailo Framework, we anticipate tensions and challenges in relation to responsible, embedded and sustainable design ( Goodyear-Smith *et al.*, 2015)- which relates to the first tension about change or initiative fatigue. Given the highly constrained economic climate ( The Health Foundation, 2022), it is necessary and essential that what gets designed locally can be implemented and sustained within existing and available local resources and assets - be these financial, human (e.g., through existing workforces) - or within existing infrastructures (physical/environmental, economic or social).

Subsequent papers and results from the developmental and realist evaluation will report on further learning, findings and how the Kailo Framework evolves.

## Data Availability

No data is associated with this article.

## References

[ref-1] Adikari S Keighran H Sarbazhosseini H : Embed Design Thinking in Co-Design for Rapid Innovation of Design Solutions. *International Conference of Design, User Experience, and Usability.* 2016; [Accessed 17 Aug. 2023]. 10.1007/978-3-319-40409-7_1

[ref-2] Alderwick H Hutchings A Mays N : A Cure for Everything and nothing? Local Partnerships for Improving Health in England. *BMJ.* 2022;378: e070910. 10.1136/bmj-2022-070910 35788447 PMC9273030

[ref-3] Alegría M NeMoyer A Falgàs Bagué I : Social Determinants of Mental Health: Where We Are and Where We Need to Go. *Curr Psychiatry Rep.* 2018;20(11): 95. 10.1007/s11920-018-0969-9 30221308 PMC6181118

[ref-4] Anderson-Carpenter KD Watson-Thompson J Jones MD : Improving Community Readiness for Change through Coalition Capacity building: Evidence from a Multi-site Intervention. *J Community Psychol.* 2017;45(4):486–499. 10.1002/jcop.21860 28458405 PMC5407193

[ref-5] Arthur MW Hawkins JD Pollard JA : Measuring Risk and Protective Factors for Substance Use, Delinquency, and Other Adolescent Problem Behaviors. *Eval Rev.* 2002;26(6):575–601. 10.1177/0193841X0202600601 12465571

[ref-6] Axford N Hobbs T : Getting the Measure of Child Health and Development Outcomes (1): a Method for Use in Children’s Services Settings. *Child Ind Res.* 2010;4(1):59–80. 10.1007/s12187-010-9074-2

[ref-7] Baumann SL : The Limitations of Evidenced-Based Practice. *Nurs Sci Q.* 2010;23(3):226–230. 10.1177/0894318410371833 20558652

[ref-8] Beames JR Kikas K O’Gradey-Lee M : A New Normal: Integrating Lived Experience Into Scientific Data Syntheses. *Front Psychiatry.* 2021;12: 763005. 10.3389/fpsyt.2021.763005 34777064 PMC8585932

[ref-9] Betancourt TS Rubin-Smith JE Beardslee WR : Understanding locally, culturally, and Contextually Relevant Mental Health Problems among Rwandan Children and Adolescents Affected by HIV/AIDS. *AIDS Care.* 2011;23(4):401–412. 10.1080/09540121.2010.516333 21271393 PMC3057405

[ref-10] Black L Humphrey N Panayiotou M : Mental Health and Well-being Measures for Mean Comparison and Screening in Adolescents: an Assessment of Unidimensionality and Sex and Age Measurement Invariance. *Assessment.* 2023; 10731911231158623. 10.1177/10731911231158623 36864693 PMC10822075

[ref-11] Boaz A Davies H Fraser A : What Works Now? *Policy Press.* Policy Press,2019; [Accessed 6 Jul. 2023]. Reference Source

[ref-12] Bolton M Moore I Ferreira A : Community Organizing and Community health: Piloting an Innovative Approach to Community Engagement Applied to an Early Intervention Project in South London. *J Public Health (Oxf).* 2016;38(1):115–121. 10.1093/pubmed/fdv017 25724610 PMC4750521

[ref-13] Braun V Clarke V Hayfield N : Thematic Analysis. *Handbook of Research Methods in Health Social Sciences.* 2019;843–860. 10.1007/978-981-10-5251-4_103

[ref-14] Brown EC Hawkins JD Arthur MW : Prevention Service System Transformation Using *Communities That Care*. *J Community Psychol.* 2011;39(2):183–201. 10.1002/jcop.20426 23606774 PMC3629975

[ref-15] Burgess R Dedios Sanguineti MC Maldonado-Carrizosa D : Using Participatory Action Research to Reimagine Community Mental Health Services in Colombia: a mixed-method Study Protocol. *BMJ Open.* 2022;12(12): e069329. 10.1136/bmjopen-2022-069329 36549743 PMC9772630

[ref-16] Burkhardt JT Schröter DC Magura S : An Overview of evidence-based Program Registers (EBPRs) for Behavioral Health. *Eval Program Plann.* 2015;48:92–99. 10.1016/j.evalprogplan.2014.09.006 25450777 PMC4413923

[ref-17] Castelpietra G Knudsen AKS Agardh EE : The Burden of Mental disorders, Substance Use Disorders and self-harm among Young People in Europe, 1990–2019: Findings from the Global Burden of Disease Study 2019. *Lancet Reg Health Eur.* 2022;16: 100341. 10.1016/j.lanepe.2022.100341 35392452 PMC8980870

[ref-18] Catalano RF Fagan AA Gavin LE : Worldwide Application of Prevention Science in Adolescent Health. *Lancet.* 2012;379(9826):1653–1664. 10.1016/S0140-6736(12)60238-4 22538180 PMC4398056

[ref-19] Chilenski SM Gayles JG Luneke A : Understanding Community- and System-capacity Change over time: a Close Look at Changing Social Capital in Evidence2Success Communities. *J Community Psychol.* 2023;51(7):2989–3011. 10.1002/jcop.23034 36971011 PMC10940032

[ref-20] Chinman M Hunter SB Ebener P : The Getting to Outcomes Demonstration and Evaluation: an Illustration of the Prevention Support System. *Am J Community Psychol.* 2008;41(3–4):206–224. 10.1007/s10464-008-9163-2 18278551 PMC2964843

[ref-21] Cicchetti D Rogosch FA : Equifinality and Multifinality in Developmental Psychopathology. *Dev Psychopathol.* 1996;8(4):597–600. 10.1017/s0954579400007318

[ref-22] Clarke RE Briggs J Armstrong A : Socio-materiality of trust: co-design with a Resource Limited Community Organisation. *CoDesign.* 2021;17(3):258–277. 10.1080/15710882.2019.1631349

[ref-23] Cohen M : A Systemic Approach to Understanding Mental Health and Services. *Soc Sci Med.* 2017;191:1–8. 10.1016/j.socscimed.2017.08.037 28881215

[ref-24] Compton MT Shim RS : The Social Determinants of Mental Health. *FOCUS.* 2015;13(4):419–425. 10.1176/appi.focus.20150017

[ref-25] Connors EH Arora P Curtis L : Evidence-Based Assessment in School Mental Health. *Cogn Behav Pract.* 2015;22(1):60–73. 10.1016/j.cbpra.2014.03.008

[ref-26] Crowley DM Greenberg MT Feinberg ME : The Effect of the PROSPER Partnership Model on Cultivating Local Stakeholder Knowledge of Evidence-Based Programs: a Five-Year Longitudinal Study of 28 Communities. *Prev Sci.* 2011;13(1):96–105. 10.1007/s11121-011-0250-5 21986990 PMC3350746

[ref-27] Darabi N Hosseinichimeh N : System Dynamics Modeling in Health and Medicine: a Systematic Literature Review. *Syst Dyn Rev.* 2020;36(1):29–73. 10.1002/sdr.1646

[ref-28] Dryden-Palmer KD Parshuram CS Berta WB : Context, Complexity and Process in the Implementation of evidence-based innovation: a Realist Informed Review. *BMC Health Serv Res.* 2020;20(1): 81. 10.1186/s12913-020-4935-y 32013977 PMC6998254

[ref-29] Duncan F Baskin C McGrath M : Community Interventions for Improving Adult Mental health: Mapping Local Policy and Practice in England. *BMC Public Health.* 2021;21(1): 1691. 10.1186/s12889-021-11741-5 34530779 PMC8444510

[ref-30] Fagan AA Bumbarger BK Barth RP : Scaling up Evidence-Based Interventions in US Public Systems to Prevent Behavioral Health Problems: Challenges and Opportunities. *Prev Sci.* 2019;20(8):1147–1168. 10.1007/s11121-019-01048-8 31444621 PMC6881430

[ref-31] Fagan AA Hawkins JD Farrington DP : Communities That Care: Building Community Engagement and Capacity to Prevent Youth Behavior Problems. Oxford Scholarship Online. Oxford University Press,2018. 10.1093/oso/9780190299217.001.0001

[ref-32] Faust HS Menzel T : Prevention vs. Treatment: What’s the Right Balance?New York: Oxford University Press,2011; [Accessed 10 Jul. 2023]. 10.1093/acprof:oso/9780199837373.001.0001

[ref-34] Frerichs L Kim M Dave G : Stakeholder Perspectives on Creating and Maintaining Trust in Community-Academic Research Partnerships. *Health Educ Behav.* 2017;44(1):182–191. 10.1177/1090198116648291 27230272 PMC6051524

[ref-35] Fricker M : Epistemic Injustice: Power and the Ethics of Knowing. academic.oup.com. Oxford University Press,2007; [Accessed 17 Aug. 2023]. 10.1093/acprof:oso/9780198237907.001.0001

[ref-36] Fried EI Robinaugh DJ : Systems All the Way down: Embracing Complexity in Mental Health Research. *BMC Med.* 2020;18(1): 205. 10.1186/s12916-020-01668-w 32660482 PMC7359484

[ref-37] Gamble J : A Developmental Evaluation Primer.Montreal: The J.W. McConnell Family Foundation,2008; [Accessed 17 Aug. 2023]. Reference Source

[ref-38] Glasgow RE Emmons KM : How Can We Increase Translation of Research into Practice? Types of Evidence Needed. *Annu Rev Public Health.* 2007;28(1):413–433. 10.1146/annurev.publhealth.28.021406.144145 17150029

[ref-39] Glenn J Kamara K Umar ZA : Applied Systems thinking: a Viable Approach to Identify Leverage Points for Accelerating Progress Towards Ending Neglected Tropical Diseases. *Health Res Policy Syst.* 2020;18(1): 56. 10.1186/s12961-020-00570-4 32493485 PMC7268457

[ref-40] Goodyear-Smith F Jackson C Greenhalgh T : Co-design and Implementation research: Challenges and Solutions for Ethics Committees. *BMC Med Ethics.* 2015;16(1): 78. 10.1186/s12910-015-0072-2 26573410 PMC4647576

[ref-41] Green F Lowther K Simpson D : RAPID-CYCLE DESIGN AND TESTING WHAT, WHY, AND HOW?Dartington Service Design Lab,2021; [Accessed 6 Jul. 2023]. Reference Source

[ref-42] Groark C Sclare I Raval H : Understanding the Experiences and Emotional Needs of Unaccompanied asylum-seeking Adolescents in the UK. *Clin Child Psychol Psychiatry.* 2011;16(3):421–442. 10.1177/1359104510370405 21317184

[ref-43] Gunnell D Kidger J Elvidge H : Adolescent Mental Health in Crisis. *BMJ.* 2018;361: k2608. 10.1136/bmj.k2608 29921659

[ref-44] Hannigan B Coffey M : Where the Wicked Problems are: the Case of Mental Health. *Health Policy.* 2011;101(3):220–227. 10.1016/j.healthpol.2010.11.002 21126794

[ref-45] Harvey AG Gumport NB : Evidence-based Psychological Treatments for Mental disorders: Modifiable Barriers to Access and Possible Solutions. *Behav Res Ther.* 2015;68(68):1–12. 10.1016/j.brat.2015.02.004 25768982 PMC4395546

[ref-46] HM Government: Life Sciences Vision (HTML). GOV.UK,2021;1–63. [Accessed 5 Jul. 2023]. Reference Source

[ref-47] Hodges S Ferreira K Israel N : “If We’re Going to Change Things, It Has to Be Systemic:” Systems Change in Children’s Mental Health. *Am J Community Psychol.* 2012;49(3–4):526–537. 10.1007/s10464-012-9491-0 22302435

[ref-48] Hughes MC Spana E Cada D : Developing a Needs Assessment Process to Address Gaps in a Local System of Care. *Community Ment Health J.* 2022;58(7):1329–1337. 10.1007/s10597-022-00940-y 35072911 PMC8785380

[ref-49] Hulme A Thompson J Brown A : The Need for a Complex Systems Approach in Rural Health Research. *BMJ Open.* 2022;12(10): e064646. 10.1136/bmjopen-2022-064646 36192093 PMC9535183

[ref-50] Jagosh J : Realist Synthesis for Public Health: Building an Ontologically Deep Understanding of How Programs Work, for Whom, and in Which Contexts. *Annu Rev Public Health.* 2019;40(1):361–372. 10.1146/annurev-publhealth-031816-044451 30633712

[ref-51] Joseph-Williams N Edwards A Elwyn G : Power imbalance prevents shared decision making. *BMJ.* 2014;348(7): g3178. 10.1136/bmj.g3178 25134115

[ref-121] Kennedy L March A Harris J : How does Kailo work to improve adolescent mental health? A developmental realist evaluation protocol.(Forthcoming).

[ref-52] Knapp M Wong G : Economics and Mental health: the Current Scenario. *World Psychiatry.* 2020;19(1):3–14. 10.1002/wps.20692 31922693 PMC6953559

[ref-53] Kohn R Saxena S Levav I : The Treatment Gap in Mental Health care. *Bull World Health Organ.* 2004;82(11):858–866. 15640922 PMC2623050

[ref-54] Lee A : Saving Normal: An Insider Revolts Against Out-of-Control Psychiatric Diagnosis, DSM-5, Big Pharma, and the Medicalization of Ordinary Life. By Dr Allen Frances. Wililam Morrow. 2013. US$20.86 (hb). 336pp. ISBN: 9780062229250. *Br J Psychiatry.* Cambridge University Press,2014;204(1):85–86. 10.1192/bjp.bp.113.134965

[ref-55] Lennon M : The State of Children’s Mental Health Services 2020/21. 2021;1–16. Reference Source

[ref-56] Leventon J Fleskens L Claringbould H : An Applied Methodology for Stakeholder Identification in Transdisciplinary Research. *Sustain Sci.* 2016;11(5):763–775. 10.1007/s11625-016-0385-1 30174742 PMC6106094

[ref-57] Local Government Association and NHS Clinical Commissioners: Localising Decision making: a Guide to Support Effective Working across neighbourhood, Place and System | Local Government Association. www.local.gov.uk, Local Government Association,2020;1–10. [Accessed 6 Jul. 2023]. Reference Source

[ref-58] Lund C Brooke-Sumner C Baingana F : Social Determinants of Mental Disorders and the Sustainable Development Goals: a Systematic Review of Reviews. *Lancet Psychiatry.* 2018;5(4):357–369. 10.1016/S2215-0366(18)30060-9 29580610

[ref-59] Manzano A : The Craft of Interviewing in Realist Evaluation. *Evaluation.* 2016;22(3):342–360. 10.1177/1356389016638615

[ref-60] Manzano A : Conducting Focus Groups in Realist Evaluation. * Evaluation (Lond).* 2022;28(4):406–425. 10.1177/13563890221124637 36212730 PMC9530522

[ref-61] Marmot M : Health Equity in England: the Marmot Review 10 Years on.The Health Foundation,2020; [Accessed 5 Jul. 2023]. Reference Source 10.1136/bmj.m693 32094110

[ref-62] Mc-Daid D Park AL : The Economic Case for Investing in the Prevention of Mental Health Conditions in the UK. www.mentalhealth.org.uk.2022; [Accessed 5 Jul. 2023]. Reference Source

[ref-63] McCabe E Amarbayan MM Rabi S : Youth Engagement in Mental Health research: a Systematic Review. *Health Expect.* 2023;26(1):30–50. 10.1111/hex.13650 36385452 PMC9854331

[ref-64] McKercher KA : Beyond Sticky Notes: Co-Design for Real: Mindsets, Methods and Movements. Beyond Sticky Notes,2020;1–225. Reference Source

[ref-65] McMillan SS King M Tully MP : How to Use the Nominal Group and Delphi Techniques. *Int J Clin Pharm.* 2016;38(3):655–62. 10.1007/s11096-016-0257-x 26846316 PMC4909789

[ref-66] Meadows DH : Thinking in Systems: a Primer. Chelsea Green Publishing,2008; [Accessed 17 Aug. 2023]. Reference Source

[ref-67] Metz A Jensen T Farley A : Building Trusting Relationships to Support implementation: a Proposed Theoretical Model. *Front Health Serv.* 2022;2: 894599. 10.3389/frhs.2022.894599 36925800 PMC10012819

[ref-68] Mullen EJ Streiner DL : The Evidence for and against Evidence-Based Practice. *Brief Treatment and Crisis Intervention.* 2004;4(2):111. 10.1093/brief-treatment/mhh009

[ref-69] Muñoz RF Mrazek PJ Haggerty RJ : Institute of Medicine Report on Prevention of Mental disorders: Summary and commentary. *Am Psychol.* 1996;51(11):1116–1122. 10.1037//0003-066x.51.11.1116 8937259

[ref-70] National Research Council (US), Institute of Medicine (US) Committee on the Prevention of Mental Disorders, Substance Abuse Among Children, Youth, Young Adults: Research Advances, Promising Interventions: Preventing Mental, Emotional, and Behavioral Disorders among Young People: Progress and Possibilities. Washington (DC): National Academies Press (US),2009; [Accessed 6 Jul. 2023]. 10.17226/12480

[ref-71] Newlove-Delgado T Marcheselli F Williams T : Mental Health of Children and Young People in England 2022 - Wave 3 Follow up to the 2017 Survey. NDRS,2022; [Accessed 5 Jul. 2023]. Reference Source

[ref-72] NHS England: NHS England» NHS England proposes new mental health access standards. www.england.nhs.uk.2021; [Accessed 5 Jul. 2023]. Reference Source

[ref-73] NHS England, NICE, NCCMH: Implementing the Early Intervention in Psychosis Access and Waiting Time Standard: Guidance. 2016. Reference Source

[ref-74] Noubani A Diaconu K Ghandour L : A Community-based System Dynamics Approach for Understanding Factors Affecting Mental Health and Health Seeking Behaviors in Beirut and Beqaa Regions of Lebanon. *Global Health.* 2020;16(1): 28. 10.1186/s12992-020-00556-5 32228648 PMC7106684

[ref-75] Nyein KP Caylor JR Duong NS : Beyond positivism: toward a Pluralistic Approach to Studying ‘real’ Teams. *Organ Psychol Rev.* 2020;10(2):87–112. 10.1177/2041386620915593

[ref-76] O’Brien J Fossey E Palmer VJ : A Scoping Review of the Use of Co-design Methods with Culturally and Linguistically Diverse Communities to Improve or Adapt Mental Health Services. *Health Soc Care Community.* 2021;29(1):1–17. 10.1111/hsc.13105 32686881

[ref-77] O’Leary P Tsui M : Lived experience: a Constant Companion for the Social Work Relationship. *Int Soc Work.* 2022;65(6):1075–1077. 10.1177/00208728221138677

[ref-78] OECD: Corporate Governance - OECD. Oecd.org.2019; [Accessed 6 Jul. 2023]. Reference Source

[ref-79] Oesterle S Kuklinski MR Hawkins JD : Long-Term Effects of the Communities That Care Trial on Substance Use, Antisocial Behavior, and Violence through Age 21 Years. *Am J Public Health.* 2018;108(5):659–665. 10.2105/AJPH.2018.304320 29565666 PMC5888048

[ref-80] Office for National Statistics: Drug misuse in England and Wales - Office for National Statistics. www.ons.gov.uk.2022; [Accessed 5 Jul. 2023]. Reference Source

[ref-81] Pailthorpe BC : Emergent Design. *The International Encyclopedia of Communication Research Methods.* 2017;1–2. 10.1002/9781118901731.iecrm0081

[ref-82] Patel V Goodman A : Researching Protective and Promotive Factors in Mental Health. *Int J Epidemiol.* 2007;36(4):703–707. 10.1093/ije/dym147 17646185

[ref-83] Pawson R Tilley N : Realistic Evaluation.SAGE Publications Ltd,1997. Reference Source

[ref-84] Pratt B : Inclusion of Marginalized Groups and Communities in Global Health Research Priority-Setting. *J Empir Res Hum Res Ethics.* 2019;14(2):169–181. 10.1177/1556264619833858 30866721

[ref-85] Public Health England: Community-centred practice: Applying All Our Health. GOV.UK,2018; [Accessed 5 Jul. 2023]. Reference Source

[ref-86] Raine R Fitzpatrick R de Pury J : Challenges, Solutions and Future Directions in Evaluative Research. *J Health Serv Res Policy.* 2016;21(4):215–216. 10.1177/1355819616664495 27522068

[ref-87] Rycroft-Malone J Seers K Titchen A : What Counts as Evidence in evidence-based practice? *J Adv Nurs.* 2004;47(1):81–90. 10.1111/j.1365-2648.2004.03068.x 15186471

[ref-88] Rycroft-Malone J Burton C Wilkinson J : Collaboration between Researchers and practitioners: How and Why Is It More Likely to Enable implementation? a Rapid Realist Review.3rd ed. Southampton, UK: NIHR Journals Library, www.ncbi.nlm.nih.gov.2015;1–165. [Accessed 6 Jul. 2023]. Reference Source

[ref-89] Salam Z Odenigbo O Newbold B : Systemic and Individual Factors That Shape Mental Health Service Usage among Visible Minority Immigrants and Refugees in Canada: a Scoping Review. *Adm Policy Ment Health.* 2022;49(4):552–574. 10.1007/s10488-021-01183-x 35066740

[ref-120] Santana de Lima E Preece C Potter K : A community-based approach to identifying and prioritising young people’s mental health needs in their local communities. *Journal of Research Involvement and Engagement.* 2023. 10.1186/s40900-023-00510-w PMC10666450 37996912

[ref-90] Saul JE Willis CD Bitz J : A time-responsive Tool for Informing Policy making: Rapid Realist Review. *Implement Sci.* 2013;8(1): 103. 10.1186/1748-5908-8-103 24007206 PMC3844485

[ref-91] Schiavo R : What Is True Community Engagement and Why It Matters (now More than ever). *J Commun Healthc.* 2021;14(2):91–92. 10.1080/17538068.2021.1935569

[ref-92] Schröder-Pfeifer P Talia A Volkert J : Developing an Assessment of Epistemic trust: a Research Protocol. *Res Psychother.* 2018;21(3): 330. 10.4081/ripppo.2018.330 32913771 PMC7451362

[ref-93] Seixas BV Regier DA Bryan S : Describing Practices of Priority Setting and Resource Allocation in Publicly Funded Health Care Systems of high-income Countries. *BMC Health Serv Res.* 2021;21(1): 90. 10.1186/s12913-021-06078-z 33499854 PMC7839200

[ref-94] Sharma S Walton M Manning S : Social Determinants of Health Influencing the New Zealand COVID-19 Response and Recovery: a Scoping Review and Causal Loop Diagram. *Systems.* 2021;9(3):52. 10.3390/systems9030052

[ref-95] Shearn K Allmark P Piercy H : Building Realist Program Theory for Large Complex and Messy Interventions. *Int J Qual Methods.* 2017;16(1): 160940691774179. 10.1177/1609406917741796

[ref-96] Shidhaye R : Implementation Science for Closing the Treatment Gap for Mental Disorders by Translating Evidence Base into practice: Experiences from the PRIME Project. *Australas Psychiatry.* 2015;23(6 Suppl):35–37. 10.1177/1039856215609771 26634667

[ref-97] Siokou C Morgan R Shiell A : Group Model building: a Participatory Approach to Understanding and Acting on Systems. *Public Health Res Pract.* 2014;25(1): e2511404. 10.17061/phrp2511404 25828443

[ref-98] Spoth R Redmond C Shin C : PROSPER community-university partnership delivery system effects on substance misuse through 6 1/2 years past baseline from a cluster randomized controlled intervention trial. *Prev Med.* 2013;56(3–4):190–196. 10.1016/j.ypmed.2012.12.013 23276777 PMC3632253

[ref-99] Spoth R Redmond C Shin C : PROSPER Delivery of Universal Preventive Interventions with Young adolescents: long-term Effects on Emerging Adult Substance Misuse and Associated Risk Behaviors. *Psychol Med.* 2017;47(13):2246–2259. 10.1017/S0033291717000691 28399955 PMC5963524

[ref-100] Spuerck I Stankovic M Fatima SZ : International Youth Mental Health Case Study of Peer Researchers’ Experiences. *Res Involv Engagem.* 2023;9(1): 33. 10.1186/s40900-023-00443-4 37189172 PMC10186639

[ref-101] Stansfield J Cavill N Marshall L : Using Complex Systems Mapping to Build a Strategic Public Health Response to Mental Health in England. *J Public Ment Health.* 2021;20(4):286–297. 10.1108/JPMH-10-2020-0140

[ref-102] Substance Abuse and Mental Health Services Administration (SAMHSA): A Guide to SAMHSA’s Strategic Prevention Framework Acknowledgments. 2019. Reference Source

[ref-103] The Health Foundation: UK Spent around a Fifth Less than European Neighbours on Health Care in Last Decade. www.health.org.uk. 2022; [Accessed 6 Jul. 2023]. Reference Source

[ref-104] Thomas F Hansford L Ford J : Moral Narratives and Mental health: Rethinking Understandings of Distress and Healthcare Support in Contexts of Austerity and Welfare Reform. *Palgrave Commun.* 2018;4(1). 10.1057/s41599-018-0091-y

[ref-105] Tindall RM Ferris M Townsend M : A First-hand Experience of Co-design in Mental Health Service design: Opportunities, challenges, and Lessons. *Int J Ment Health Nurs.* 2021;30(6):1693–1702. 10.1111/inm.12925 34390117

[ref-106] Tuomela R : The Philosophy of Sociality. *Oxford University PressNew York eBooks.* Oxford University Press,2007. 10.1093/acprof:oso/9780195313390.001.0001

[ref-107] Ungar M Theron L : Resilience and Mental health: How Multisystemic Processes Contribute to Positive Outcomes. *Lancet Psychiatry.* 2020;7(5):441–448. 10.1016/S2215-0366(19)30434-1 31806473

[ref-108] Vangen S Huxham C : Nurturing Collaborative Relations. *J Appl Behav Sci.* 2003;39(1):5–31. 10.1177/0021886303039001001

[ref-109] Westhorp G : A Methods Lab Publication: Realist Impact Evaluation: An Introduction. ODI.org. Australian Government Department of Foreign Affairs and Trade,2014; [Accessed 15 Sep. 2023]. Reference Source

[ref-110] Wilkins CH : Effective Engagement Requires Trust and Being Trustworthy. *Med Care.* 2018;56 Suppl 10 Suppl 1(10 Suppl 1):S6–S8. 10.1097/MLR.0000000000000953 30015725 PMC6143205

[ref-111] Wolstenholme EF : Towards the Definition and Use of a Core Set of Archetypal Structures in System Dynamics. *Syst Dyn Rev.* 2003;19(1):7–26. 10.1002/sdr.259

[ref-112] Wong G Westhorp G Manzano A : RAMESES II Reporting Standards for Realist Evaluations. *BMC Med.* 2016;14(1): 96. 10.1186/s12916-016-0643-1 27342217 PMC4920991

[ref-113] World Health Organisation: Department of Mental Health and Substance Dependence: Mental Health Evidence Review: Prevention and Promotion in Mental Health.Geneva: World Health Organisation,2002; [Accessed 10 Jul. 2023]. Reference Source

