# Supplementary material for: Kailo: a systemic approach to addressing the social determinants of young people’s mental health and wellbeing at the local level
Source: Wellcome Open Res. 2023 Nov 13;8:524. [Version 1] doi: 10.12688/wellcomeopenres.20095.1 (PMC11126905; doi:10.12688/wellcomeopenres.20095.1)
Supplement: Peer review history

# Reviewer report: Reviewer response for version 1

**DOI:** 10.21956/wellcomeopenres.22254.r73611

**Published:** 2024-05-25

**Authors:** Tessa Reardon

**Affiliations:** 1 Departments of Experimental Psychology and Psychiatry, University of Oxford, Oxford, England, UK

The authors present a convincing case for the need for systemic approaches that address the social determinants of young people’s mental health and wellbeing at a local level. They clearly articulate the implications of focusing only on treatment at the expense of preventive efforts, and the need to consider the complex, interconnected influences on mental health and how this varies across contexts. Limitations with existing prevention frameworks are presented, including limitations related to underpinning data, the absence of young people’s voices, and neglecting local practice and innovation. The paper moves on to present aims to create, implement and refine the Kailo prevention framework that seeks to address limitations with previous frameworks.  The guiding principles of Kailo, together with inputs, activities, intended outcomes and indicators of success across three phases of the programme are clearly spelled out.  An overview of initial evaluation plans is provided and expected challenges and responses helpfully discussed.  It may help to clarify how evaluation is considered in the early phases of the programme. For example, does co-designing systemic responses incorporate co-designing evaluation of these responses?  Adolescent mental health outcomes are mentioned in later parts of the paper – it would help to clarify and specify earlier in the paper if the initiative focuses on a specific age range.

Does the article adequately reference differing views and opinions?

Yes

Are all factual statements correct, and are statements and arguments made adequately supported by citations?

Yes

Is the Open Letter written in accessible language?

Yes

Where applicable, are recommendations and next steps explained clearly for others to follow?

Yes

Is the rationale for the Open Letter provided in sufficient detail?

Yes

Reviewer Expertise:

child mental health; school-based interventions

I confirm that I have read this submission and believe that I have an appropriate level of expertise to confirm that it is of an acceptable scientific standard.

# Reviewer report: Reviewer response for version 1

**DOI:** 10.21956/wellcomeopenres.22254.r69990

**Published:** 2024-05-25

**Authors:** Josefien Breedvelt, Sasha Arya

**Affiliations:** 1 University of Amsterdam, The Netherlands; Centre for Urban Mental Health, University of Amsterdam, Amsterdam, The Netherlands; 2 King's College London, London, England, UK

This is an open letter which details the research and design initiative 'Kailo' which combines research and design to inform better embedded and sustainable strategies to address social determinants of young people's mental health at a local level.

 Overall, it provides an overview of an innovative and relevant approach which highlights crucial issues as working with local partners and involving young people in systems change activities when supporting and delivering preventative interventions and changes for mental health that address social determinants.

**Background**

Need and inequalities

 - The references and sources provided in the first two paragraphs of the Needs and inequalities section are England/UK (and one EU) focused, We think it would be helpful to clarify that the statements given specifically relate to the area cited (e.g. UK/England/EU). Or if the authors wish to make a more generic statement, we suggest to seek international references to support their statement (especially in the second paragraph).

 - If space allows, it would benefit us to know which aspects of mental health the authors would like to address (perhaps by a definition of mental health and wellbeing or at minimum highlight that it is all mental health conditions and wellbeing that Kailo is concerned about, and/or that outcomes will be co-determined with young people and local partners (for example)).

Existing frameworks for understanding local needs and guiding prevention efforts

 - "These prevention frameworks have, in some contexts, demonstrated positive impacts on outcomes ( Brown et al., 2011; Crowley et al., 2011; Oesterle et al., 2018; Spoth et al., 2017)." - please can the authors specify on which outcomes there was an improvement and whether these effects were sustainable. At minimum we  would recommend the authors highlight that it is "some" outcomes, as the references cover mainly substance use and knowledge outcomes. It may also be helpful to mention the specific positive impacts in each citation (e.g. Oesterle et al. (2018) found a reduction in substance use, antisocial behaviour and violence, whereas Crowley et al. (2011) found increases in expert knowledge of evidence-based interventions).

**Accessibility**

 - Where possible, providing definitions to concepts introduced in the manuscript would support the reader’s understanding and would ensure this article is accessible to a wider audience. This includes concepts mentioned, such as: “place-based”, “power mapping”, “design thinking ideation”, “epistemic trust” and “summative impact evaluation”. 

 - Regarding summative impact evaluation it may be helpful to add some examples / definitions of approaches used and how such an impact evaluation may be co-designed.

 - Some wording in the abstract could be simplified, for instance this sentence could be shortened: “…manifest or may be influenced at the local level, impeding the design of contextually nuanced preventative approaches”

Does the article adequately reference differing views and opinions?

Yes

Are all factual statements correct, and are statements and arguments made adequately supported by citations?

Partly

Is the Open Letter written in accessible language?

Partly

Where applicable, are recommendations and next steps explained clearly for others to follow?

Not applicable

Is the rationale for the Open Letter provided in sufficient detail?

Yes

Reviewer Expertise:

Breedvelt: Mental health prevention, children and young people's mental health. Arya: Young people's perspectives on preventative interventions, mental health prevention, children and young people's mental health

We confirm that we have read this submission and believe that we have an appropriate level of expertise to confirm that it is of an acceptable scientific standard, however we have significant reservations, as outlined above.

# Reviewer report: Reviewer response for version 1

**DOI:** 10.21956/wellcomeopenres.22254.r81451

**Published:** 2024-05-24

**Authors:** Abigail Thomson

**Affiliations:** 1 Centre for Psychiatry and Mental Health, Queen Mary University of London, London, England, UK

This paper introduces Kailo, a novel prevention framework designed to support local community and public system partnerships to design and implement local and contextually nuanced preventative strategies to improve children's and young people’s mental health and wellbeing.

 The authors provide a strong rationale for their framework, highlighting several limitations of current mental health treatment and research for young people, noting an overreliance on mono-causal assumptions and narrowly defined health outcomes. They frame young people’s mental health and well-being as a ’systems issue’, emphasising the role of wider and interacting social determinants. Further, they suggest that despite advancements in treatment responses, issues with funding, resources and capacity hamper these efforts and exacerbate inequality in access to mental health support (a crucial social determinant of population mental health). The authors describe the need for a more nuanced local perspective, exploring how the social determinants of mental health are manifest at the micro level, in addition to considering macro-systemic influence (e.g. poverty, housing). Further, they describe the need for better preventative support that more meaningfully elevates youth and community voices and balances evidence-informed practice with local innovation and co-design. Greater consideration could be given here to those groups of young people who are most underserved by current approaches, whose voices are underrepresented, and for whom the ‘systems’ described may have a greater impact on their mental health and well-being.

 Overall, the Kailo Framework is described as aiming to improve, at the local level, youth mental health and wellbeing outcomes via the design and implementation of preventative approaches that address contextually relevant social determinants of health. It is described as operating across three phases: (i) Early Discovery - building local partnerships and shared priorities (ii) Deeper discovery and Co-Design - youth-centred co-design methods (iii) Prototyping, Implementation and Testing - embedding designs into local infrastructures. The framework's modularity and flexibility are highlighted, alongside the importance of evaluation and future development. The authors could consider describing how their approach plans to better serve the many small and successful community health projects that operate under the radar of formal evaluations and those that are dynamic by nature (i.e. participatory methods) where changes often occur long after formal evaluation.

 The initial and anticipated challenges of the Kailo framework are well considered, and the authors outline some of the steps needed going forward to address these challenges. Attention is given to understanding how different types of evidence manifest within a local and community context and how this may be at odds with current recognised approaches. Further discussion could be given around plans to address this, beyond developing a shared understanding, particularly for those stakeholders or community partners most impacted by systemic inequalities that are contributing their voice to the project.

Does the article adequately reference differing views and opinions?

Yes

Are all factual statements correct, and are statements and arguments made adequately supported by citations?

Yes

Is the Open Letter written in accessible language?

Yes

Where applicable, are recommendations and next steps explained clearly for others to follow?

Yes

Is the rationale for the Open Letter provided in sufficient detail?

Yes

Reviewer Expertise:

Community-based participatory research; Youth engagement/involvement in mental health research; Community-based implementation; Digital interventions; Transdiagnostic approaches to treatment/prevention of adolescent psychopathology

I confirm that I have read this submission and believe that I have an appropriate level of expertise to confirm that it is of an acceptable scientific standard.

# Reviewer report: Reviewer response for version 1

**DOI:** 10.21956/wellcomeopenres.22254.r69992

**Published:** 2023-12-15

**Authors:** Jo Henderson

**Affiliations:** 1 Centre for Addiction and Mental Health, Toronto, Canada

The authors of this paper present a compelling case for re-imagining conventional approaches to addressing child and youth mental health problems. They argue that improved mental health and wellbeing for children and youth will not be achieved through individual-level treatment of mental health disorders alone, though investment is argued to be warranted here. Instead, the authors argue that significant investment must be made in designing, testing, and scaling effective, locally determined population-level prevention and promotion approaches. The authors convincingly articulate the limitations of current approaches and amplify the important message that existing single-factor causal explanations for deteriorating child and youth mental health, such as those based on social media, redefining mental ill health, or expanding help-seeking options are inadequate. Instead, given the importance of the social determinants of mental health in understanding child and youth mental health, the authors argue that child and youth mental health must be considered a ‘systems issue’. While acknowledging the important role of macro-system consideration, the authors argue for attention to micro/local social determinants, delineating the value of exploring local manifestations of the social determinants of mental health and building local preventative approaches.

 In particular, the authors describe the creation of ‘Kailo’, a new systemic prevention framework designed to address, at a local level, the social determinants of mental health impacting children and youth. Kailo builds upon existing prevention frameworks to be more inclusive of youth and community voices, local innovation and systemic considerations. The authors describe Kailo as operating in three distinct but interconnected phases: 1. Early discovery (building partnerships, learning, prioritizing); 2. Deeper discovery and co-design (structured youth-centered co-design emphasizing equity, evidence and systems approaches); and, 3. Prototyping, implementing, and testing (in local infrastructures).   The authors provide concrete aims, inputs, pre-requisites, activities, intended outcomes, and indicators of success for each phase, providing the reader with a foundational map for implementing the Kailo framework. An addition that could strengthen the Kailo framework as presented in this article is greater attention to capturing the relationship components that are centered in the model. It is unlikely that critical relationship factors will be captured by numbers of engagement activities or diversity of participants. These are necessary but not likely sufficient for achieving Kailo’s goals. Instead, quality or strength of relationship, as well as youth or community member perceptions of empowerment could be considered.  In terms of principles, it is surprising that commitment to measurement/understanding impact is not articulated as a key principle.

 The authors describe current efforts to test the Kailo framework in two distinct UK communities and an intention to revise Kailo to Kailo v2.0 based on learnings from early implementation and developmental evaluation. Notably, the methods described align very well with the articulated commitments to centering youth and community voices. Consideration of a Learning Health Systems approach (e.g., Menear et al., 2019) in the discussion of implementation and learning over time may also strengthen the discussion. Future work by the Kailo team will include scaling to additional sites and a summative evaluation. Initial and anticipated challenges are well articulated. Additional discussion of how to address the issue of who holds power in defining evidence and how evidence is defined would be informative, as would even a very brief discussion of the optimal skills and capabilities required for a successful Kailo team.

Does the article adequately reference differing views and opinions?

Yes

Are all factual statements correct, and are statements and arguments made adequately supported by citations?

Yes

Is the Open Letter written in accessible language?

Yes

Where applicable, are recommendations and next steps explained clearly for others to follow?

Yes

Is the rationale for the Open Letter provided in sufficient detail?

Yes

Reviewer Expertise:

Clinical and health services research in child and youth mental health; youth, family and community engagement

I confirm that I have read this submission and believe that I have an appropriate level of expertise to confirm that it is of an acceptable scientific standard.

## References

[1] : A framework for value-creating learning health systems. *Health Res Policy Syst* .2019;17(1) : 10.1186/s12961-019-0477-3 79 10.1186/s12961-019-0477-3 31399114 PMC6688264
